# Partial Discharges and Noise Discrimination Using Magnetic Antennas, the Cross Wavelet Transform and Support Vector Machines

**DOI:** 10.3390/s20113180

**Published:** 2020-06-03

**Authors:** Fabio Muñoz-Muñoz, Armando Rodrigo-Mor

**Affiliations:** Electrical Sustainable Energy, Delft University of Technology, 2600 GA Delft, The Netherlands; A.RodrigoMor@tudelft.nl

**Keywords:** partial discharges, GIS, magnetic antenna, machine learning, feature selection, wavelet analysis, noise separation, support vector machines.

## Abstract

This paper presents a wavelet analysis technique together with support vector machines (SVM) to discriminate partial discharges (PD) from external disturbances (electromagnetic noise) in a GIS PD measuring system based on magnetic antennas. The technique uses the Cross Wavelet Transform (XWT) to process the PD signals and the external disturbances coming from the magnetic antennas installed in the GIS compartments. The measurements were performed in a high voltage (HV) GIS containing a source of PD and common-mode external disturbances, where the external disturbances were created by an electric dipole radiator placed in the middle of the GIS. The PD were created by connecting a needle to the main conductor in one of the GIS compartments. The cross wavelet transform and its local relative phase were used for feature extraction from the PD and the external noise. The features extracted formed linearly separable clusters of PD and external disturbances. These clusters were automatically classified by a support vector machine (SVM) algorithm. The SVM presented an error rate of 0.33%, correctly classifying 99.66% of the signals. The technique is intended to reduce the PD false positive indications of the common-mode signals created by an electric dipole. The measuring system fundamentals, the XWT foundations, the features extraction, the data analysis, the classification algorithm, and the experimental results are presented.

## 1. Introduction

Nowadays, partial discharge (PD) measurements are essential for assessing the condition of high-voltage equipment because of its intrinsic ability to detect incipient defects in the insulation system. In general, the PD measurement systems are based on electrical techniques, in which the PD are measured by electrical sensors such as coupling capacitors, ultra high frequency (UHF) antennas, high frequency current transformers (HFCT), and magnetic sensors. PD measurements are fundamental in the monitoring of high voltage (HV) GIS due to its high capabilities to detect in-service failures related to defects in the insulation system [1].

Among all the methods to measure PD in HV-GIS, the system based on UHF antennas is the most widely used because it is less affected by noise [1], that is mainly concentrated at low frequencies. The UHF method uses capacitive couplers tuned at the ultra-high frequency range (300–3 GHz), which are placed in the dielectric windows present in the GIS compartments. The UHF antennas are shielded from external electromagnetic interferences because the GIS enclosure acts as a Faraday cage. This shielding guarantees a low background noise and high sensitivity for the PD monitoring, consolidating the application of UHF antennas as the standard method for on-line PD monitoring in AC and DC GIS. However, the UHF measuring system is vulnerable to the noise coming from radio stations, mobile phone signals, and TV transmitters, and, in consequence, some UHF measuring systems include an external gating (UHF antenna placed outside the GIS compartments) which helps to identify this external noise.

In a GIS, at the UHF range, the PD signals suffer significant attenuation and distortion [2,3,4,5,6,7], causing a substantial reduction of the PD detection sensitivity with the distance between the PD source and the sensor. This disadvantage is solved by deploying several UHF along the GIS in order to reliably monitor all the GIS components. Moreover, at the UHF range, the pulses measured and the PD charge are uncorrelated, indicating that it is not possible to estimate the apparent charge in pC [1].

**Figure 1:**
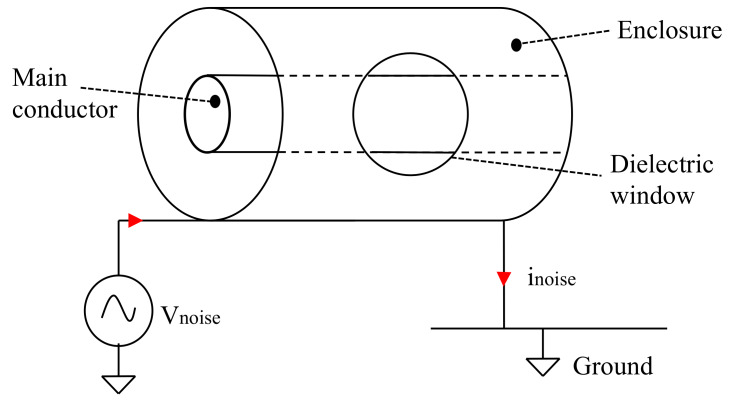
Common-mode induction in the GIS.

Due to the GIS dimensions, from kHz to hundreds of MHz, the PD current travels as in a transmission line (transverse electromagnetic (TEM) mode of propagation), suffering significantly less attenuation and distortion than at the UHF range. However, the PD measurements are strongly affected by the noise coming from power electronics, the power grid, or, in general, external electromagnetic disturbances. The advances in post-processing and acquisition techniques allowed us to develop a new PD measuring system for HV GIS [8], in which the PD currents are captured by magnetic antennas tuned at the high frequency and very high frequency (HF-VHF) range (1 MHz–300 MHz). The magnetic antenna consists of two half-circle loops, producing two signal outputs per pulse measured [8]. The magnetic antennas are placed in the dielectric windows in such a way that a current signal, traveling inside the GIS in the TEM mode, causes a symmetric response in the two magnetic antenna loops. This new measuring system is capable of estimating the PD apparent charge, and detecting PD pulses below 5 pC [9]. Nevertheless, the magnetic antennas are vulnerable to the ingress of external disturbances, affecting the measuring system reliability. For instance, the common-mode signals, represented in Figure 1, may affect the measuring system based on magnetic antennas, causing PD false positives. The $V_{noise}$ represents a differential voltage that appears between the ground and the GIS enclosure. $V_{noise}$ creates a common-mode current ($i_{noise}$) that flows in the outer surface of the GIS enclosure. The $i_{noise}$ reaches the dielectric window and could be picked up by a magnetic antenna placed at the dielectric window.

**Figure 2:**
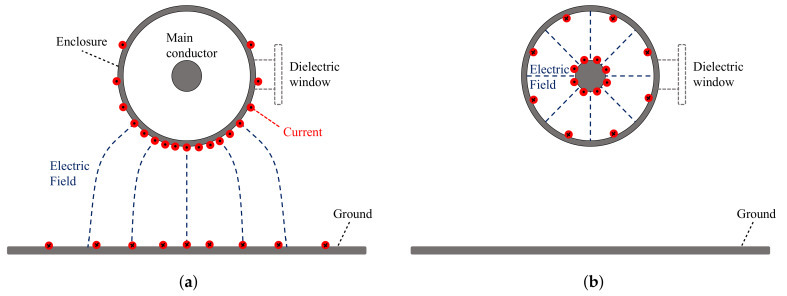
Cross-section propagation: (**a**) common-mode signal, (**b**) partial discharge (PD) pulse inside a GIS compartment.

A cross-section representation of the propagation of the common-mode signal can be seen in Figure 2a, where the current flows in the GIS enclosure and the ground. In this parallel circle-plate waveguide, the current does not flow homogeneously distributed in the surface of the enclosure, and an electromagnetic wave travels in higher-modes of propagation (transverse electric (TE) or transverse magnetic (TM)) than the TEM mode. In contrast, the PD pulses, shown in Figure 2b, travel in a coaxial waveguide, which causes a homogeneous distribution of the current in the enclosure and an electromagnetic wave that travels in the TEM mode in the frequencies below the UHF range. Be aware that the parallel circle-plate waveguide presented is a particular example; therefore, different waveguide configurations are expected for the common-mode signals. Due to this difference in the propagation modes, a PD pulse causes a symmetric output in the two magnetic antenna loops because the PD current flows homogeneously distributed in the GIS enclosure and around the dielectric window. On the other hand, a common-mode signal should produce an asymmetric output in the magnetic antenna because the common-mode current does not necessarily travel homogeneously distributed in the enclosure and around the dielectric window.

For denoising PD measurements, the wavelet transform has been used in several studies [10,11,12,13,14,15,16] because it is capable of transforming the signals in the time and frequency domain, helping in the analysis of aperiodic signals with irregular and transition features, such as the PD [17]. However, these wavelet-based denoising techniques do not tackle the problem of pulse-type external interferences, which are the primary source of noise contamination for the magnetic antennas. In general, these external disturbances are pulse-shaped interferences from power electronics, external corona discharges, electrical pulses from switching operations, lightings, etc. In [18,19,20], separation techniques for pulse-type signals have been developed, in which the energy, waveform, and frequency spectrum of the signals are used to build up clusters for the identification of the nature of the signals. Nevertheless, noise pulses with time-frequency characteristics similar to the PD signals, could be misclassified by these techniques. For external disturbances having waveforms similar to the PD signals, the Phase Resolved PD (PRPD) pattern is a very effective tool to cluster PD and noise contamination, but its application is limited to PD under AC, and, therefore, for PD under DC additional tools for noise separation are needed.

In this study, a technique for separation of PD signals from external disturbances is proposed for the magnetic antennas, where the external disturbances are created by an electric dipole radiator. The technique uses the cross wavelet transform (XWT) analysis for the symmetry evaluation of the signals measured by the magnetic antennas, where this evaluation results in a set of features which are used to train support vector machine (SVM) classification algorithm for the automatic recognition of PD signals and the common-mode external disturbances. The technique provides a tool for the discrimination of common-mode external disturbances in PD measurements based on magnetic antennas. The common-mode external disturbances were mimicked by an electric dipole radiator and transmitted and picked up by the GIS acting as an antenna. In PD measurements, the common-mode signals are usually unwelcome guests, which are caused by radiofrequency interferences such as lightings, switching operations, radio, TV, radar systems, thermostat-controlled or motor-controlled devices [21]. For building up the technique, PD measurements were performed in a HV GIS, in which PD signals and external disturbances were recorded. The magnetic antenna, the XWT analysis, the experimental test, the features extraction and analysis, the SVM algorithm, and the separation results are presented.

The structure of the paper is described as follows: Section 2 briefly introduces the magnetic antenna fundamentals. Section 3 shows the setup description and the experimental results. Section 4 introduces the cross wavelet analysis and its application to the magnetic antenna signals. The feature extraction technique using the cross wavelet transform and its analysis are presented in Section 5. The SVM classifier, trained for the noise separation, is studied in Section 6. Finally, Section 7 presents the conclusions of the paper.

## 2. Magnetic Antenna Fundamentals

**Figure 3:**
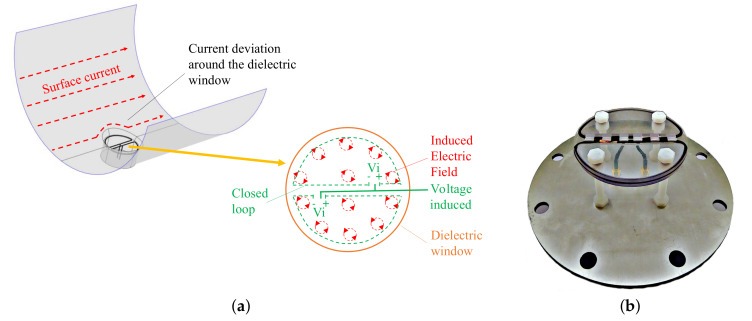
Magnetic antenna: (**a**) PD pulse propagation inside a GIS compartment. (**b**) Magnetic antenna.

A PD pulse propagates along the GIS compartments in the TEM mode, creating a surface current in the GIS enclosure, as is shown in Figure 3a. This surface current $i_{PD}$ travels homogeneously distributed in the enclosure until it reaches a dielectric window that causes a deviation of $i_{PD}$ around the window. The deviation in the window produces a time-varying magnetic field in the surface formed by the dielectric window [9]. A magnetic antenna was made up of two shielded loops to pick up this time-varying magnetic field, as is shown in Figure 3b. Each loop is made of a coaxial cable RG179 wound in a half-circle shape. The magnetic antenna is mounted in the dielectric windows destined for the UHF couplers, and it is fixed to a conductive mounting plate that keeps the internal Faraday cage capabilities of the GIS enclosure. Further details of the magnetic antenna dimensions and its electrical circuit parameters can be found in [9]. The magnetic antenna loops (labeled as top and bottom loop) measure both the magnetic field and the electric field produced by the PD pulse. To get rid of the electric field and only measure the magnetic field, the outer loop of the coaxial cable is grounded in one side and left floating in the other to prevent short-loop currents and to provide electric field shielding. The inner conductor of the coaxial cable forms the measuring loop and is connected to a parallel of 2 × 0.056 F decoupling capacitors, which is connected to a commercial transimpedance amplifier model Femto HCA-400M-5K-C. The decoupling capacitor blocks the DC feedback loop between the amplifier and the magnetic antenna. The antenna loops are set in such a way that a PD current traveling inside the GIS compartments ideally induce a voltage with the same amplitude in each loop, but with opposite polarity.

## 3. Setup Description and Experimental Results

**Figure 4:**
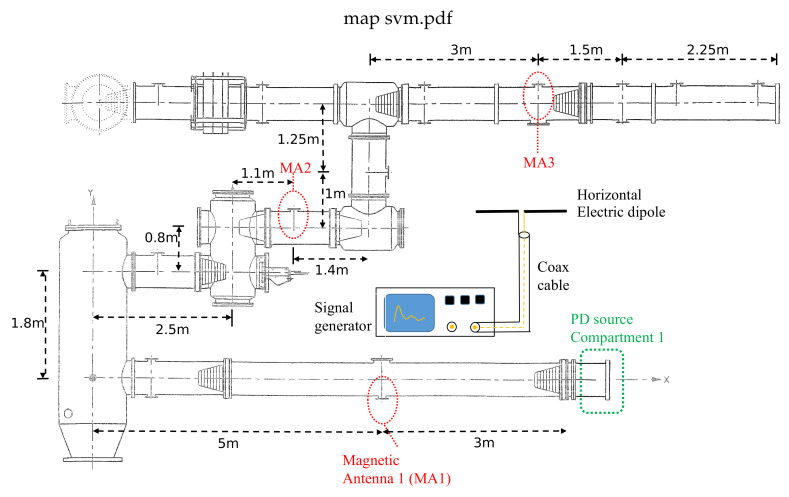
Test setup.

The test setup consists of a real HV GIS at the High Voltage Laboratory of TU Delft, a HV DC source, three magnetic antennas, a PD defect to create PD pulses, an electric dipole radiator to produce the common-mode external disturbances, and a digital oscilloscope as acquisition unit. The 380 kV GIS has multiple spacers, a T-joint branch, a switchgear, a bushing, an L-branch connection, a disconnector switch, and eight dielectric windows for PD monitoring. Three of the eight dielectric windows were used by magnetic antennas at the locations highlighted in Figure 4, where each antenna was named as MA1, MA2 and MA3.

Inside the GIS, corona discharges were produced by a needle connected to the main conductor at the compartment 1. To trigger the PD activity, −15 kV DC were applied to the GIS. The electric dipole radiator consisted of two copper conductors of equal length (0.5 m each one) oriented in a horizontal position to the ground plane (at 2 m above it). The electric dipole radiator was placed in the middle of the GIS, and it was connected to a fast pulse generator to create the external disturbances which were picked up by the PD measuring system. The signals measured by the magnetic antennas were transmitted through identical coaxial cables to the digital oscilloscope, which was set at 1.25 GS/s. Each signal was recorded individually in a frame with a record length of 2.05 s. The three magnetic antennas were simultaneously sampled by the oscilloscope.

Three tests were conducted, as is described in Table 1. In the first test, only PD signals were acquired because the external disturbances were switched off. In the second test, the PD were absent, and only external disturbances were collected. In the third test, both PD and external disturbances were recorded by the measuring system.

**Figure 5:**
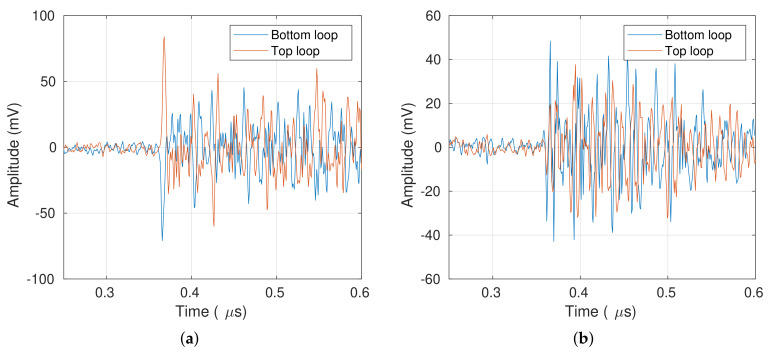
Signals measured by the MA1: (**a**) PD signal. (**b**) External disturbance.

Figure 5a presents a PD pulse recorded by the magnetic antenna MA1 during Test 1. The magnetic antenna signals show that the first peak of both loops appears around the same time (0.36 s), have opposite polarities, similar magnitudes, and a mirror-like symmetry. All the signals recorded in Test 1 have the same traits described before. It is essential to consider that a perfect mirror-like symmetry cannot be achieved due to manufacturing differences between loops and small antenna misalignments with respect to the dielectric window.

Figure 5b shows an external disturbance acquired during Test 2. Unlike the PD signals, the external disturbance does not have the mirror-like symmetry, and both signals start with the same polarity. Once again, all the signals acquired in Test 2 do not have the mirror-like symmetry of the PD signals. It shows that these external disturbances do not travel in the TEM mode inside the GIS.

## 4. Cross Wavelet Transform (XWT) Analysis

Taking into account that the magnetic antenna has two signal outputs, the combination of both signals may provide useful information about the nature of the waveform recorded and, thus, it may help to discriminate whether the signals come from a PD event or an external disturbance. For this analysis, the correlation and the trend analysis can provide the significance of the relationships between the two signals recorded. However, these tools may not detect a correlation between both signals because they are set in opposite polarity (phase-shifted $180^{\circ }$) and, then the signals may appear uncorrelated [22]. The cross-correlation and the cross-spectral analysis can detect the phase shift, but only in stationary signals. For analyzing aperiodic signals with irregular and transition features, the most suitable tool is the cross wavelet transform (XWT) because it exposes regions with high common power and reveals the local relative phase between both signals [23].

A wavelet is a small wave of limited duration that has zero mean. The sinusoids used in the Fourier transform are periodic, smooth, predictable, and are the most suitable option at describing constant-frequency signals (periodic signals) [24]. On the other hand, wavelets are irregular, of limited duration, and non-symmetrical. Therefore, wavelets are the best option for analyzing signals with anomalies, non-periodic signals, pulses, and, in general, events that start and stop within the signal [24].

The Fourier transform consists of decomposing a signal into sinusoidal waves of different frequencies, the wavelet transform decomposes the signal into a shifted and scaled version of the mother wavelet [12]. The continuous wavelet transform (CWT) of a signal $x_{n}$ (n=1,…,N) is defined as the convolution of $x_{n}$ with the scaled and normalized wavelet [25].

(1) $$W_{n}^{x}\left(s\right)=\sqrt{\frac{\delta t}{s}}\sum_{n^{\prime }=1}^{N}x_{n^{\prime }}\psi_{0}\left[\left(n^{\prime }-n\right)\frac{\delta t}{s}\right]$$

where *s* is the scale, δt is the time step, and $\psi_{0}$ is the mother wavelet. The CWT of $x_{n}$ is a s×N matrix of wavelet coefficients, i.e., for each scale *s* there is a corresponding time series(n=1,…,N). The mother wavelet $\psi_{0}$ is in general complex, and therefore the CWT is also complex [26]. The CWT can be seen as a mathematical tool that transforms a time-domain signal $x_{n}$ into the time-scale domain. The CWT is an appropriate tool for analyzing non-periodic signals, anomalies, or transients because it transforms the signal to a time-frequency representation. However, when it comes to analyzing the correlation between two signals, the Cross Wavelet Transform (XWT) is the right tool for finding links between two signals. The XWT is a measure of similarity between two waveforms [27], exposing regions with high common power and revealing information about the phase relationship. The XWT of two signals $x_{n}$ and $y_{n}$ is defined as:

(2) $$W_{n}^{xy}\left(s\right)=W_{n}^{x}\left(s\right)W_{n}^{y*}\left(s\right)$$

where the $W_{n}^{x}$ and $W_{n}^{y}$ are the CWT of $x_{n}$ and $y_{n}$ respectively, and the operator ∗ indicates the complex conjugate. If the mother wavelet $\psi_{0}$ is complex, the XWT is a complex matrix than can be decomposed into amplitude and phase using the following equation:

(3) $$W_{n}^{xy}\left(s\right)=\left|W_{n}^{xy}\left(s\right)\right|e^{j\cdot arg(W_{n}^{xy}\left(s\right))}$$

where $|W^{xy}|$ is the XWT modulus (magnitude) and $arg(W^{xy})$ denotes the XWT argument (phase). The XWT modulus represents the cross-amplitudes of $x_{n}$ and $y_{n}$[22], whereas the XWT argument provides an estimation of the local phase difference $\Delta \phi_{n}^{xy}\left(s\right)$ between the two signals for each time-scale coefficient [28]. In other words, $\Delta \phi_{n}^{xy}\left(s\right)$ is the local relative phase between both signals [25]. Another useful tool is the wavelet coherence, which measures how coherent the cross wavelet transform is in time-frequency space [28]. The wavelet coherence is defined in Equation (4).

(4) $$R_{n}^{2}\left(s\right)=\frac{|S\left(W_{n}^{xy}\left(s\right)\right){|}^{2}}{S(|W_{n}^{x}{\left(s\right)|}^{2})S(|W_{n}^{y}\left(s\right){|}^{2})}$$

where *S* is a smoothing operator in time and scale. In [25], the smoothing operator is presented as:

(5) $$S\left(W\right)=S_{scale}\left(S_{time}\left(W_{n}\left(s\right)\right)\right)$$

where $S_{scale}$ represents the smoothing along the wavelet scales and $S_{time}$ the smoothing in time.

### XWT and the Magnetic Antenna

Section 3 shows that the PD pulses and the external disturbances both present a correlation between the signals in the magnetic antenna loops. However, in the PD pulse, the correlation would have a phase shift of $180^{\circ }$ because the signals have opposite polarity. Keeping this in mind, the XWT may check the correlation and its relative phase between the signals acquired by the magnetic antenna loops. If a PD event is acquired by both loops, the XWT will show a high common power around the PD event and a relative phase around $180^{\circ }$. On the other hand, an external disturbance will also present a high common power around the pulse, but with a different phase shift. To corroborate this hypothesis, a wavelet analysis, consisting of four steps, is applied to signals measured by the magnetic antennas. The analysis is illustrated using the signals plotted in Figure 5.

In the first step, the electrical pulse measured by both loops (x(t) and y(t)) are decomposed in the time-frequency domain by the CWT, where the complex wavelet Morlet is chosen as mother wavelet. Each signal has N=2564samples and a time step equal to $\delta t=\frac{1}{1.25\;GS/s}\;=\;0.8\;ns$. The signals were decomposed in 89 scales, ranging from 1 MHz to 625 MHz. The CWT of each signal results in a 89 × 2564 matrix.

**Figure 6:**
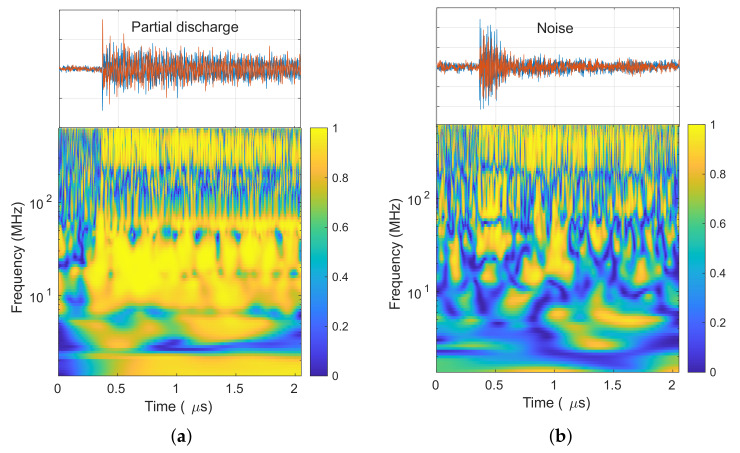
Wavelet coherence: (**a**) PD signal. (**b**) External disturbance.

In the second step, the XWT is calculated using Equation (2). Then, using Equation (4) the wavelet coherence is obtained and plotted in Figure 6a for the PD pulse in Figure 5a. Figure 6b shows the wavelet coherence for the external disturbance depicted in Figure 5b. The wavelet coherence of both signals shows that the signals have high common power along the pulses, but there is not a clear distinction of the real nature of each signal. Therefore, further steps are needed to clear up the wavelet coherence by adding the phase information.

In the third step, the local relative phase $\Delta \phi_{n}^{xy}\left(s\right)$ is computed. After this, the elements in the $\Delta \phi_{n}^{xy}\left(s\right)$ matrix that have a relative phase higher than $160^{\circ }$ are selected, whereas the elements having a relative phase lower than $160^{\circ }$ are ignored, as is shown in Equation (6); it should be considered that a “perfect” opposite polarity means $180^{\circ }$ local phase, but because of the discretization of the signals, the noise, and the manufacturing differences between loops, a lower local phase than $180^{\circ }$ would be expected.

(6) $$ind\Delta \phi_{n}^{xy}\left(s\right)=\left\{\begin{array}{cc} 1 & \mathrm{if}\;\Delta \phi_{n}^{xy}\left(s\right)\geq 160^{\circ }\\ 0 & \mathrm{if}\;\Delta \phi_{n}^{xy}\left(s\right)<160^{\circ } \end{array}\right.$$

In the final step, the $ind\Delta \phi_{n}^{xy}\left(s\right)$ is multiplied (element-wise) by the wavelet coherence, as is presented in Equation (7). In $R160_{n}^{2}\left(s\right)$ only the elements having a local phase higher or equal than $160^{\circ }$ are kept, whereas the rest is annulled. The $R160_{n}^{2}\left(s\right)$ results in a 89 × 2564 matrix for each pulse event.

(7) $$R160_{n}^{2}\left(s\right)=R_{n}^{2}\left(s\right)\cdot ind\Delta \phi_{n}^{xy}\left(s\right)$$

**Figure 7:**
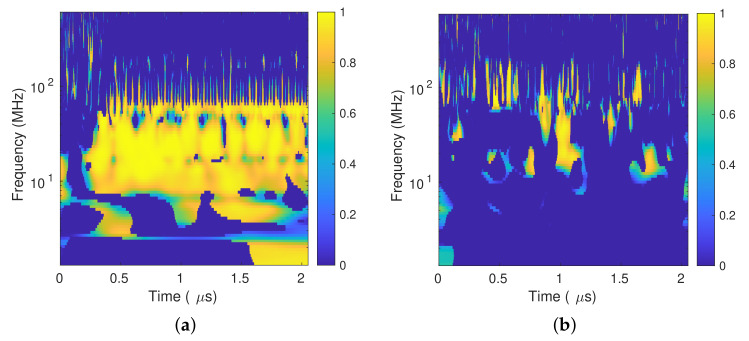
Wavelet coherence for a $\Delta \phi_{n}^{xy}\left(s\right)\geq 160^{\circ }$: (**a**) PD signal. (**b**) External disturbance.

Figure 7a depicts the wavelet coherence $R160_{n}^{2}\left(s\right)$ for the PD pulse in Figure 5, which shows a high and constant correlation between the signals along the PD duration; between 7 MHz to 70 MHz and starting around 0.3 s; there is a yellow stripe indicating a high correlation between both signals. On the other hand, Figure 7b shows the $R160_{n}^{2}\left(s\right)$ for the external disturbance, which does not present a clear and constant correlation along the signal duration.

## 5. Features Extraction

When the local relative phase is higher than $160^{\circ }$, the $R160_{n}^{2}\left(s\right)$ indicates how coherent the cross wavelet transform is in the time domain for each wavelet scale. A way of weighting the coherence of each scale is by computing the mean of each scale in $R160_{n}^{2}\left(s\right)$. By applying the mean to each scale, the original data set goes from a 89 × 2564 matrix to a 89 elements vector, reducing the dimensionality of the wavelet coherence $R160_{n}^{2}\left(s\right)$. In Equation (8), the mean of the wavelet coherence is defined. The reduction in the dimension of the data helps us to analyze the features by using tools such as the principal components analysis (PCA). Each signal measured by the magnetic antennas is represented by a 89 elements vector or 89 features, containing the mean value of each scale in the wavelet coherence.

(8) $$\hat{R}\left(s\right)=\frac{1}{N}\sum_{n=1}^{N}R160_{n}^{2}\left(s\right)$$

Equations (7) and (8) are computed for all the tests performed, resulting in three data sets. Data set 1 (300signals×89scales) corresponds to the Test 1, Data set 2 (300signals×89scales) to the Test 2, and Data set 3 (3000signals×89scales) to the Test 3. The Test 1 contains 100 pulses measured simultaneously by three magnetic antennas, resulting in 300 signals recorded by the acquisition unit; the same analysis applies for Tests 2 and 3.

### Principal Components Analysis (PCA)

PCA is the process in which the principal components are computed, and the subsequent use of these components in understanding the data (learning from the data) [29]. The PCA is used to perform an exploratory analysis of the data by summarizing the data set with a smaller number of representative variables, which collectively explain the variability of the original set [29]. The PCA is a useful tool for displaying multidimensional data by reducing the data complexity.

**Figure 8:**
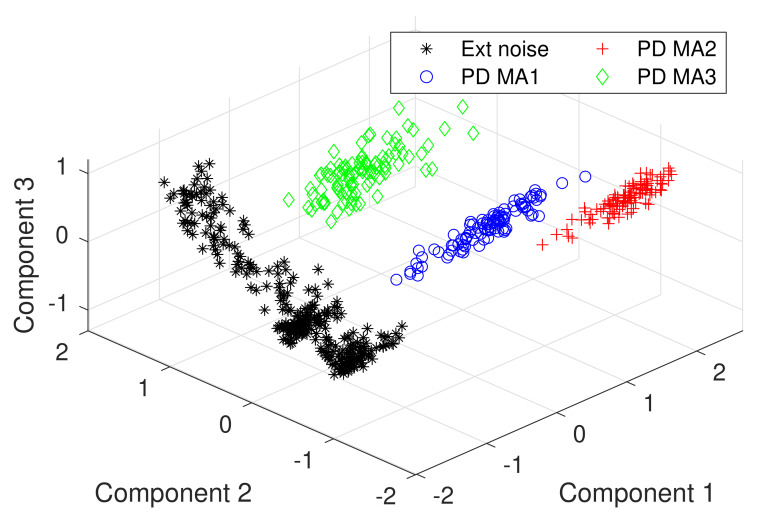
Principal components analysis (PCA) analysis to the Data sets 1 and 2.

In this study, the PCA is used to perform an exploratory analysis of the data by reducing its dimensionality; the data sets have 89-dimensions, making impossible the visual representation. The principal components of the Data sets 1 and 2 are computed, resulting in a 3-dimensional representation of the data set which can be depicted in a 3D plot. It this PCA, the first three components explain 89% of all the data set variability. The first three components are plotted in Figure 8, showing the formation of four clusters. One cluster shows the PD signals in the MA1, the second cluster groups the PD signals in the MA2, the third one represents the PD in MA3, and the fourth cluster shows the external disturbances picked up by all the magnetic antennas.

From the PCA, two conclusions pop up:

- The wavelet coherence ($R160_{n}^{2}\left(s\right)$) and its mean value ($\hat{R}\left(s\right)$) are appropriate tools to extract relevant features that help us to separate the external disturbances from the PD signals.
- The cluster formation gives way to fit a supervised classifier such as the SVM to separate the PD from the external disturbances automatically.

## 6. SVM Classifier

The support vector machine is a generalization of a simple and intuitive classifier called the maximal margin classifier, which was developed in the 1990s [29]. SVM are intended for the binary classification setting in which there are two classes [29], like the one in this study: partial discharge or external disturbance. The support vector machine’s algorithm finds the optimal separation between these two classes by computing a separating hyperplane. SVM algorithm presents low generalization error (avoid over-fitting) [30], is computationally inexpensive, and its results are easy to interpret. If the data is linearly separable, a linear SVM classifier is sufficient [30]. The data are linearly separable if there are two groups of data, and the data points are separated enough that it is possible to draw a straight line on the figure with all the points of one class on one side of the line and all the points of the other class on the other side of the line [31]. This line is called a separating hyperplane, which is a simple line in the 2D plane but evolves to a separating hyperplane in an N-dimensional plane. The hyperplane plays the role of a decision boundary, where everything on one side belongs to one class, and everything on the other side belongs to a different class [31].

**Figure 9:**
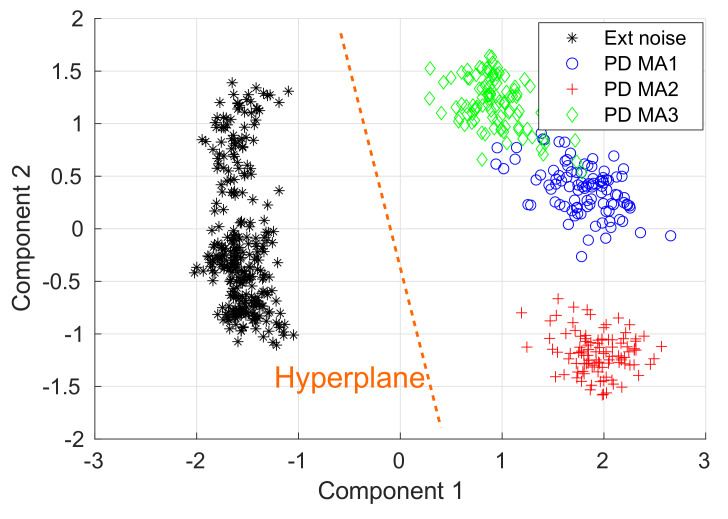
Support vector machine (SVM) proof of concept applied to the first two components of the PCA.

Returning to the classification problem of this study, it is possible to infer that the data is linearly separable because it forms two groups in each side of a hypothetical separating hyperplane, as is illustrated in Figure 9, where the two first components of the PCA conducted in Section 5 are plotted. The separating hyperplane was arbitrarily drawn in the middle of the two clusters, working as a decision boundary in which all the data at the left might be considered noise, and all the data at the right may be labeled as partial discharge. Further details of support vector machines can be found in [29,30,31].

**Figure 10:**
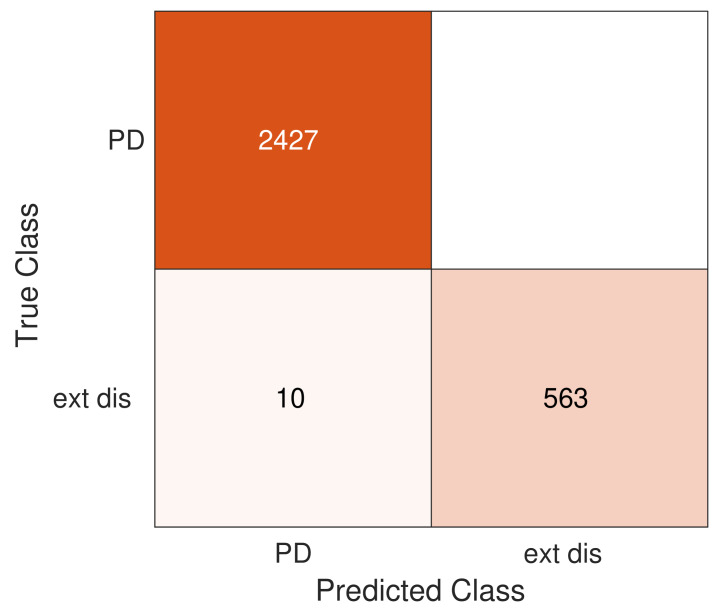
Confusion matrix.

In Section 5 it was found that the mean value of $R160_{n}^{2}\left(s\right)$ forms clear and separated clusters for each category(class): PD and external disturbance. This shows that the mean value can be used as a good clean input data for a supervised classification algorithm(classifier), which can predict the class of a new input signal. The Data sets 1 and 2 are chosen as the training set to build/train the SVM classifier. The PD in the MA1, MA2, and MA3 are merged in a unique class named partial discharge, whereas the external disturbances in the three antennas are labeled as external disturbance. All the PD in the Data set 1 are labeled as partial discharge, and the external disturbances in the Data set 2 are labeled as external disturbance. For the evaluation of the SVM classifier performance, the Data set 3 is used as a validation set. The validation set contains 3000 pulses measured by the three magnetic antennas, in which 2427 corresponds to PD pulses and the 573 remaining pulses to external disturbances. The corresponding class of each element in this data set was manually verified. A SVM classifier is built using the training set and the Gaussian kernel. The performance of the SVM classifier is tested with the validation set, getting an error rate of 0.33 %. The confusion matrix is depicted in Figure 10, indicating that 2427 PD were correctly classified as PD, 563 external disturbances were successfully labeled, and 10 external disturbances were misclassified as PD. These results show that the XWT based analysis, together with the SVM classifier, are useful tools to identify PD pulses from external disturbances. However, the classifier uses 89 features for each pulse recorded, without analyzing which of these 89 features are efficiently describing the input data and which ones are irrelevant for the classification problem. In the next section, a feature selection analysis is performed to analyze the relevance of the features

### Features Selection Using Pearson Correlation

**Figure 11:**
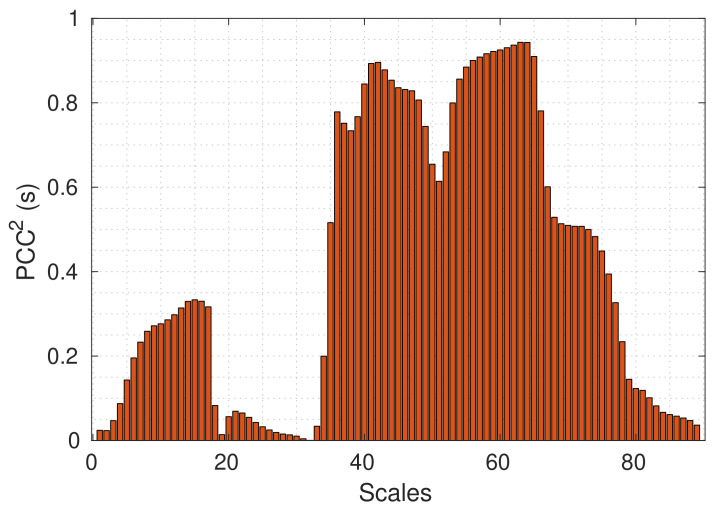
Pearson correlation coefficient.

Features selection techniques help to address the problem of reducing irrelevant and redundant features, which are a burden for the classifier and the computing time [32]. In general, feature selection helps in understanding the data, reducing computation requirements, and improving the predictor performance [32]. The focus of this feature selection study is to select a subset of the 89 features data which can efficiently describe the input data. One of these techniques is the Pearson correlation coefficient which is defined as follows:

(9) $$PCC\left(s\right)=\frac{cov(\hat{R}\left(s\right),Y)}{\sqrt{var(\hat{R}\left(s\right))*var\left(Y\right)}}$$

where $\hat{R}\left(s\right)$ is described in Equation (8), *Y* is the output class label (PD or external disturbance), cov() is the covariance, and var() is the variance. The Pearson correlation detects linear dependencies between the features(variables) and the output class. The $PCC^{2}\left(s\right)$ is used as feature ranking criterion because it enforces a ranking according to goodness of linear fit of individual features [33]. For the training set (Data sets 1 and 2), the $PCC^{2}\left(s\right)$ is computed and plotted in Figure 11.

Figure 11 shows that there is a strong linear correlation between the scales ranging from 35 to 75 and the output class, indicating that the scales subset from 35 to 75 efficiently describes the input data. It means that all the scales outside this range are irrelevant for the classification problem. This range of scales is equivalent to a frequency range from 3.7 MHz to 59 MHz. This scales subset has 41 features, implying a reduction of 48 features (less than half of the original features are needed to build a classifier). This new subset is used to build a SVM classifier, resulting in an error rate of 0.33 %, which is the same rate that the classifier got by using the original 89 features. However, the new subset resulted in a reduction of 50% in computing time.

## 7. Conclusions

The cross wavelet transform and the SVM classifier have been proven to be useful tools to build up a technique for the separation of PD pulses from common-mode external disturbances in a GIS measuring system based on magnetic antennas. The results show that this technique succeeds in the separation of PD signals from external disturbances because it presents an error rate of 0.33 %. In the validation set (3000 signals), 2427 partial discharges and 563 external disturbances were correctly identified by the SVM classifier, whereas only 10 signals were misclassified.

In the PD pulses measured by both loops in the magnetic antenna, the first peaks show a mirror-like symmetry. On the other hand, the common-mode external disturbances does not present such a symmetry. The XWT local relative phase has been proven to be an effective tool to evaluate the mirror-like symmetry of a given signal. Therefore, the XWT relative phase can be used to discriminate PD from some external disturbances.

The $R160_{n}^{2}\left(s\right)$ mean value is an appropriate tool to extract relevant features for the discrimination of PD pulses and external disturbances in PD measurements based on magnetic antennas. Additionally, the mean value reduced the dimension of the data from an 89 × 2564 matrix to a, 89 vector. The principal components analysis showed that the PD and the externals disturbances form linearly separable clusters, which gave way to deploy a SVM classifier.

The Pearson correlation indicated that the relevant scales for separating the signals are the scales ranging from 35 to 75. In the frequency domain, this range corresponds to a bandwidth from 3.7 MHz to 59 MHz.

A different source of noise could form a separate cluster that may not be easily separated by a SVM classifier. Therefore, each different source of noise should be studied and characterized before choosing the optimal classification algorithm.

## Figures and Tables

**Table 1:** Test overview.

Test	Type of Signals	Pulses Recorded
Test 1	Partial discharges	100
Test 2	External disturbances	100
Test 3	PD and External disturbances	1000 (81% are PD)
